# Negative interaction between nitrates and remote ischemic preconditioning in patients undergoing cardiac surgery: the ERIC-GTN and ERICCA studies

**DOI:** 10.1007/s00395-022-00938-3

**Published:** 2022-06-21

**Authors:** Ashraf Hamarneh, Andrew Fu Wah Ho, Heerajnarain Bulluck, Vivek Sivaraman, Federico Ricciardi, Jennifer Nicholas, Hilary Shanahan, Elizabeth A. Hardman, Peter Wicks, Manish Ramlall, Robin Chung, John McGowan, Roger Cordery, David Lawrence, Tim Clayton, Bonnie Kyle, Maria Xenou, Cono Ariti, Derek M. Yellon, Derek J. Hausenloy

**Affiliations:** 1 Institute of Cardiovascular Sciences, The Hatter Cardiovascular Institute, University College London, London, WC1E 6HX UK; 2 Department of Emergency Medicine, Singapore General Hospital, Singapore, Singapore; 3 Pre-Hospital and Emergency Research Centre, Health Services and Systems Research, Duke-NUS Medical School, Singapore, Singapore; 4 Leeds Institute of Cardiovascular and Metabolic Medicine, University of Leeds, Leeds, UK; 5 Department of Cardiology, Leeds Teaching Hospitals NHS Trust, Leeds, UK; 6 Department of Statistical Science, University College London, London, UK; 7 Clinical Trials Unit and Department of Medical Statistics, London School of Hygiene and Tropical Medicine, London, UK; 8 University College London Hospitals NHS Foundation Trust, London, UK; 9 University Hospital Southampton NHS Foundation Trust, London, UK; 10 Barts Heart Centre, King’s College London, London, UK; 11 University Hospital of Wales, Heath Park, Cardiff, CF14 4YS UK; 12 Cardiovascular and Metabolic Disorders Program, Duke-National University of Singapore Medical School, Singapore, Singapore; 13 National Heart Research Institute Singapore, National Heart Centre Singapore, Singapore, Singapore; 14 Yong Loo Lin School of Medicine, National University Singapore, Singapore, Singapore; 15 Cardiovascular Research Center, College of Medical and Health Sciences, Asia University, Taichung City, Taiwan

**Keywords:** Remote ischaemic preconditioning, Glyceryl trinitrate, Coronary artery bypass graft surgery, Cardioprotection, Ischaemia, Reperfusion injury, Nitrates

## Abstract

Remote ischaemic preconditioning (RIPC) using transient limb ischaemia failed to improve clinical outcomes following cardiac surgery and the reasons for this remain unclear. In the ERIC-GTN study, we evaluated whether concomitant nitrate therapy abrogated RIPC cardioprotection. We also undertook a post-hoc analysis of the ERICCA study, to investigate a potential negative interaction between RIPC and nitrates on clinical outcomes following cardiac surgery. In ERIC-GTN, 185 patients undergoing cardiac surgery were randomized to: (1) Control (no RIPC or nitrates); (2) RIPC alone; (3); Nitrates alone; and (4) RIPC + Nitrates. An intravenous infusion of nitrates (glyceryl trinitrate 1 mg/mL solution) was commenced on arrival at the operating theatre at a rate of 2–5 mL/h to maintain a mean arterial pressure between 60 and 70 mmHg and was stopped when the patient was taken off cardiopulmonary bypass. The primary endpoint was peri-operative myocardial injury (PMI) quantified by a 48-h area-under-the-curve high-sensitivity Troponin-T (48 h-AUC-hs-cTnT). In ERICCA, we analysed data for 1502 patients undergoing cardiac surgery to investigate for a potential negative interaction between RIPC and nitrates on clinical outcomes at 12-months. In ERIC-GTN, RIPC alone reduced 48 h-AUC-hs-cTnT by 37.1%, when compared to control (ratio of AUC 0.629 [95% CI 0.413–0.957], *p* = 0.031), and this cardioprotective effect was abrogated in the presence of nitrates. Treatment with nitrates alone did not reduce 48 h-AUC-hs-cTnT, when compared to control. In ERICCA there was a negative interaction between nitrate use and RIPC for all-cause and cardiovascular mortality at 12-months, and for risk of peri-operative myocardial infarction. RIPC alone reduced the risk of peri-operative myocardial infarction, compared to control, but no significant effect of RIPC was demonstrated for the other outcomes. When RIPC and nitrates were used together they had an adverse impact in patients undergoing cardiac surgery with the presence of nitrates abrogating RIPC-induced cardioprotection and increasing the risk of mortality at 12-months post-cardiac surgery in patients receiving RIPC.

**Supplementary Information:**

The online version contains supplementary material available at 10.1007/s00395-022-00938-3.

## Introduction

Higher risk patients are undergoing coronary revascularisation by cardiac bypass surgery, resulting in an increased risk of peri-operative myocardial injury and infarction, and worse post-operative outcomes [28, 37]. Reasons for the higher operative risk include the aging population, increasing prevalence of comorbidities (such as diabetes mellitus, hypertension, and chronic renal failure), and a growing need for concomitant valve surgery. Therefore, new treatment strategies are needed to protect the myocardium against acute ischaemia/reperfusion injury (IRI) during cardiac bypass surgery, to improve outcomes in this higher risk patient group [36].

In this regard, the phenomenon of remote ischaemic preconditioning (RIPC), in which the application of cycles of brief non-lethal ischaemia and reperfusion to an organ or tissue away from the heart, has been shown in experimental studies to protect the myocardium against acute IRI [29]. Crucially, RIPC can be applied in the clinical setting by simply inflating and deflating a pneumatic cuff placed on the upper arm or thigh to induce cycles of brief non-lethal ischaemia and reperfusion [21]. This manoeuvre has been evaluated as a cardioprotective strategy in patients undergoing cardiac bypass surgery [5, 15, 16, 32] and has been reported in a number of clinical studies [11, 34, 35], but not all [20, 30], to reduce the extent of peri-operative myocardial injury (PMI, quantified by cardiac biomarker release). However, three large multicentre randomised clinical trials failed to demonstrate any beneficial effects with RIPC on clinical endpoints [12, 17, 27]. The reasons for this are unclear but may have been due to concomitant medications administered during cardiac surgery. For example, a number of experimental [2, 4, 7, 8, 41] and clinical studies [1, 23–25] have demonstrated that propofol interferes with RIPC-induced cardioprotection, and propofol was the predominant anaesthesia used in the large neutral RIPC outcome studies [12, 17, 27].

There are experimental and clinical data suggesting that the concomitant use of nitrates may also interfere with RIPC-induced cardioprotection. Pre-treatment with the nitric oxide (NO) donor *S*-nitroso-*N*-acetylpenicillamine was shown to abrogate the infarct-limiting effects of RIPC induced by brief cycles of hind-limb ischaemia and reperfusion in a rabbit model of acute myocardial IRI [33]. More recently, chronic treatment with topical glyceryl trinitrate (GTN) was found to abrogate RIPC-induced cardioprotection in a rat model of acute myocardial IRI [10]. In the same study, it was shown that the vasculoprotective effects of RIPC on ischaemia-induced endothelial dysfunction in human volunteers was abolished in those that had received prior chronic GTN therapy [10]. In a post-hoc retrospective analysis of a small clinical study of patients undergoing cardiac bypass surgery, the cardioprotective effect of RIPC in reducing PMI was abolished in those patients administered intraoperative IV GTN [6], although in a post-hoc retrospective analysis of another cardiac surgery study this effect of IV GTN was not observed [22]. Taken together, these studies suggest a potential negative interaction between RIPC and nitrates in terms of cardioprotection.

Prophylactic intraoperative intravenous (IV) GTN is administered in some patients during cardiac surgery to control systemic blood pressure, and to vasodilate arterial grafts. There is extensive experimental data supporting the cardioprotective effects of NO [3, 10, 14, 40], but whether it is cardioprotective during cardiac surgery remains unclear [6, 18, 22]. Randomised controlled trials evaluating the cardioprotective effects of intraoperative IV GTN in patients undergoing cardiac bypass surgery are limited [18], and there are no prospective studies evaluating the interaction between nitrate use and RIPC in terms of cardioprotection in patients undergoing cardiac surgery.

Therefore, in the **E**ffect of **R**emote **I**schemic **C**onditioning and **G**lyceryl **T**ri**N**itrate (ERIC-GTN) randomised control trial, we prospectively investigated whether prophylactic intraoperative IV GTN is cardioprotective in cardiac bypass surgery, and whether its presence abrogates RIPC-induced cardioprotection [9]. We also undertook a post-hoc retrospective analysis of the previously published **E**ffect of **R**emote** I**schaemic Preconditioning on **C**linical Outcomes in **CA**BG Surgery (ERICCA) study [12], which had failed to show any beneficial effects of RIPC on clinical outcomes, to investigate for a potential negative interaction between RIPC and nitrates on perioperative myocardial injury and infarction, and all-cause and cardiovascular mortality at 12 months following cardiac bypass surgery.

## Methods

### ERIC-GTN study design

The ERIC-GTN trial was a single-centre, 2 × 2 factorial, double-blind, placebo-controlled randomised controlled trial (NCT01864252). It was designed to investigate whether intraoperative IV GTN was cardioprotective in itself, and whether its presence interferes with the cardioprotective effect of RIPC in patients undergoing cardiac surgery. It recruited patients at University College London Hospital (UCLH) and the Barts Heart Centre in the UK. Details of the study design have been published previously [9]. Trial conduct conformed to the Declaration of Helsinki 1964 as revised in 2013 and the principles of Good Clinical Practice under the oversight of University College London Hospital. Ethics approval was granted by the National Health Service Research Ethics Committee (13/LO/0980 IRAS 120058a) All participants provided written informed consent.

### Study participants

Eligible subjects were stable patients with coronary artery disease, aged ≥ 18 years, undergoing elective on-pump coronary artery bypass graft plus or minus valve surgery (CABG ± valve) with blood cardioplegia. Exclusion criteria included: history of cardiogenic shock or cardiac arrest during the current admission, pregnancy, significant peripheral arterial disease of the upper limbs, significant hepatic impairment (bilirubin > 20 mmol/L and International Normalised Ratio, INR > 2.0), significant pulmonary disease (forced expiratory volume, FEV1 < 40% predicted), severe renal failure (glomerular filtration rate, GFR < 30 mL/min/1.73 m^2^), and allergies to GTN.

All patients received premedication with oral temazepam. Anaesthetic and perioperative management were not standardised. Standard non-pulsatile cardiopulmonary bypass (CPB) was employed using a membrane oxygenator and cardiotomy suction. Following this, all coronary grafts were constructed during CPB, using blood cardioplegia. After the anastomoses of the grafts (with or without valve surgery), CPB was discontinued, and protamine was used to reverse the effect of heparin. Anaesthesia maintenance was achieved with volatile anaesthetic agents and propofol infusion. Arterial blood pressure, central venous pressure, electrocardiogram, and core temperature were continuously recorded.

### Outcomes

The primary endpoint of the study was PMI quantified by the 48-h AUC hs-cTnT. The AUC was calculated from hs-cTnT levels drawn preoperatively, and at 6-, 12-, 24- and 48-h post-surgery.

### Randomisation and blinding

Randomisation was performed via SealedEnvelope™ by an unblinded study team member who also administered the study interventions described below. The patients, anaesthetists, surgeons, intensive care unit and ward staff, and the study team members collecting and analysing the data were blinded to the treatment allocation.

#### Interventions

Patients were randomly allocated to one of the four treatment groups in a 1:1:1:1 ratio:

##### (1) Control: patients received sham RIPC and placebo IV normal saline infusion

The sham RIPC protocol was initiated after the patient had been anaesthetised and prior to surgical incision. It comprised placing one pneumatic blood pressure cuff on the upper arm and one on the thigh and applying simulated inflations of both cuffs (with the valve open to prevent actual cuff inflation) for 5 min and simulated deflations of both cuffs for 5 min, a cycle which was repeated three times in total. The placebo IV normal saline infusion was commenced on arrival at the operating theatre at a rate of 2 mL/h and stopped when the patient was taken off CPB.

##### (2) RIPC alone: patients received RIPC and placebo IV normal saline infusion

The RIPC protocol was initiated after the patient had been anaesthetised and prior to surgical incision. It comprised placing one pneumatic blood pressure cuff on the upper arm and one on the thigh. The two cuffs were simultaneously inflated to a systolic blood pressure (SBP) of 200 mmHg and left inflated for 5 min, then rapidly deflated to 0 mmHg and left uninflated for 5 min. This cycle was repeated three times in total. However, if the SBP was ≥ 185 mmHg, the cuffs were inflated to 15 mmHg above SBP instead of 200 mmHg. The placebo IV normal saline infusion was administered as in group 1.

##### (3) Nitrates alone: patients received sham RIPC and IV GTN infusion

The sham RIPC protocol was applied as in group 1. The IV GTN infusion (1 mg/mL solution) was commenced on arrival at the operating theatre at a rate of 2 mL/h and stopped when the patient was taken off CPB. The infusion rate was titrated between 2 and 5 mL/h to maintain a mean arterial pressure of 60–70 mmHg.

##### (4) RIPC + Nitrates: patients received RIPC protocol and IV GTN infusion

The RIPC protocol was applied as in group 2 and the IV GTN infusion was administered as in group 3.

Cases in which patients randomised to receive placebo IV saline infusion (treatment groups 1 and 2) needed to be given IV GTN infusion as a clinical indication for hypertension resistant to an increase in anaesthetic agents or in cases of coronary artery or graft vasospasm, were counted as crossovers to the respective IV GTN treatment group.

### Statistical analysis and sample size estimation

The sample size calculation was based on a previous trial of RIPC in CABG surgery in which subgroup analyses reported a potential beneficial effect of IV GTN on reducing the extent of PMI [6]. To detect a difference between any pair of the four treatment groups with 80% power, 5% two-sided α, and an AUC standard deviation of 21.4 μg/l, it was estimated that 50 patients will be needed in each group to observe a difference of ≥ 12 μg/l in AUC. Statistical analysis was carried out by an independent analyst. All analyses were performed using an Intention-to-treat (ITT) approach. Baseline clinical and demographic characteristics were presented as mean ± standard error of the mean (SEM) for continuous data and counts and percentage for categorical data.

Due to high levels of missing hs-cTnT data at all time-points, 50 imputed datasets were generated using multivariate imputation by chained equations (MICE), done separately for each of the four groups defined by the two treatments [39]. Variables included in the imputation model, other than the log hs-cTnT at each time point, were: gender, age, smoking status, diastolic and systolic BP, pulse, ejection fraction and length of ICU stay; CCS angina class, creatinine; prior use of aspirin, beta blockers, nitrates, diuretics, clopidogrel, insulin and metformin; prior diagnosis of MI, diabetes, peripheral arterial disease, TIA/stroke, sulphonylurea and hypercholesterolemia; previous CABG; bypass duration and number of grafts. 48-h AUC hs-cTnT was calculated for each of the 50 imputed datasets.

The primary outcome, 48-h AUC was transformed to the logarithmic scale (logAUC) as the data did not approximate normal distribution. To determine whether logAUC was different in the four arms, the primary outcome was analysed by fitting two-way ANOVA models, including a binary term for each treatment group, as well as the interaction between the two. A second no-interaction ANOVA model was fitted including only the binary term for each treatment group. We used an *F*-test to assess the statistical significance of the interaction term, by comparing the interaction model with the nested no-interaction one. All estimates from imputed datasets were pooled together using Rubin’s rules [31].

### Post-hoc analysis of the ERICCA study

We performed a non-prespecified post-hoc analysis of the previously published ERICCA multi-centre study to investigate whether the use of nitrates improves clinical outcomes, and whether the use of nitrates interacts with RIPC in terms of clinical outcomes in patients undergoing cardiac bypass surgery. The ERICCA study randomised 1612 patients undergoing on-pump CABG (plus or minus valve surgery) to receive either RIPC (comprising four 5-min upper arm cuff inflations/deflations of a pneumatic cuff placed on the upper arm) or sham RIPC (comprising four 5-min simulated upper arm cuff inflations/deflations of a pneumatic cuff placed on the upper arm) [12]. In the ERICCA trial, RIPC failed to improve clinical outcomes (cardiovascular death, non-fatal myocardial infarction, coronary revascularisation or stroke) at 12-months post-surgery.

Our post-hoc analysis included patients where CABG surgery was completed, and data were available on use of nitrates and baseline characteristics. Our primary analysis compared clinical outcomes at 12 months post-surgery in four patient groups: (1) control (sham RIPC with no nitrates); (2) RIPC alone (RIPC with no nitrates); (3) nitrates alone (sham RIPC with nitrates); (4) RIPC + Nitrates (RIPC with nitrates). Use of nitrates was classified as patients who were either treated with intraoperative IV GTN infusion and/or were using long-acting oral nitrates at baseline. A secondary analysis compared clinical outcomes in four patient groups defined according to IV GTN, regardless of use of long-acting oral nitrates: (1) control (sham RIPC without IV GTN); (2) RIPC alone (RIPC without IV GTN); (3) IV GTN alone (sham RIPC with IV GTN); (4) RIPC + GTN (RIPC with IV GTN).

The primary endpoint of this analysis was 12-month all-cause mortality. Secondary endpoints included cardiovascular mortality at 12 months, perioperative myocardial infarction (MI) and PMI (quantified by the 72-h AUC hs-cTnT). Definitions of these outcomes followed those used in the primary ERICCA analysis [12].

The models for each outcome included indicator variables for RIPC and nitrates and their interaction to allow us to test whether the effects of nitrates and RIPC when given in combination differed from the effects of nitrates and RIPC when given alone. Each model was used to estimate the effects of RIPC with and without the addition of nitrates. Although the allocation of RIPC was randomised, the use of nitrates was not randomly assigned. To address this potential source of bias, results were presented without adjustment and with adjustment for EuroSCORE, body mass index (BMI), previous myocardial infarction, and diabetes by including these as additional predictor variables.

Mortality and cardiovascular mortality were analysed using Cox proportional hazards with stratification by study site and censoring at the date of death, loss to follow-up, withdrawal from the study or at 12 months. Kaplan–Meier curves showing cumulative mortality and cardiovascular mortality were produced by the four groups defined by RIPC and nitrate use.

Due to high levels of missing data of hs-cTnT, multiple imputation was performed before analysis of 72-h AUC hs-cTnT. 50 imputed datasets were generated using Multivariate Imputation by Chained Equations (MICE), done separately for the RIPC and sham control groups. The variables in the imputation model were: log hs-cTnT at each time point (baseline, 6, 12, 24, 48, 72 h); gender, age, smoking status; baseline EuroSCORE, CCS angina class, LVEF class, natural logarithm creatinine, BMI; prior use of aspirin, beta blockers, long lasting oral nitrates, diuretics, clopidogrel and metformin; prior diagnosis of MI, diabetes, and hypercholesterolaemia; bypass duration; use of IV GTN during surgery; number of grafts; post-surgical requirement for cardiac pacing, and post-surgical acute kidney injury; study site; and cardiovascular death within 12 months of surgery. 72-h AUC hs-cTnT was calculated for each of the 20 imputed datasets.

72-h AUC hs-cTnT was transformed to the logarithmic scale (logAUC) as this more closely followed a normal distribution. It was analysed using a mixed effect linear regression model with study site included as a random effect. Estimates from imputed datasets were pooled together using Rubin’s rules.

Perioperative myocardial infarction was analysed using mixed effect logistic regression with study site included as a random effect.

For all statistical analyses of the ERIC-GTN and ERICCA studies, significance level α was set at 0.05 and two-sided 95% confidence intervals (CI) are calculated. All analyses were performed using STATA version 16 (College Station, TX, USA) or above.

## Results

### ERIC-GTN study

**Fig. 1:**
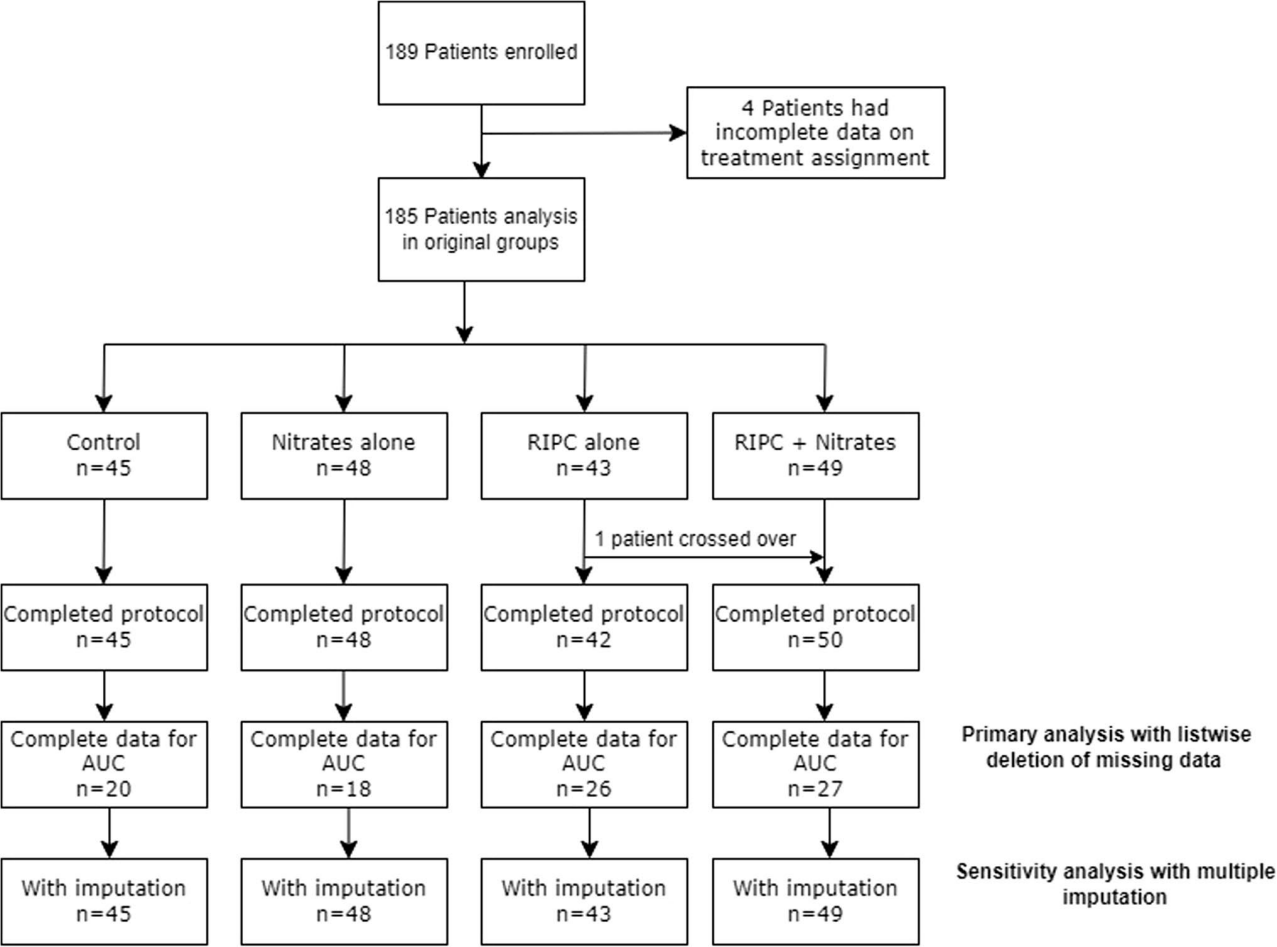
ERIC-GTN study patient flow diagram

**Fig. 2:**
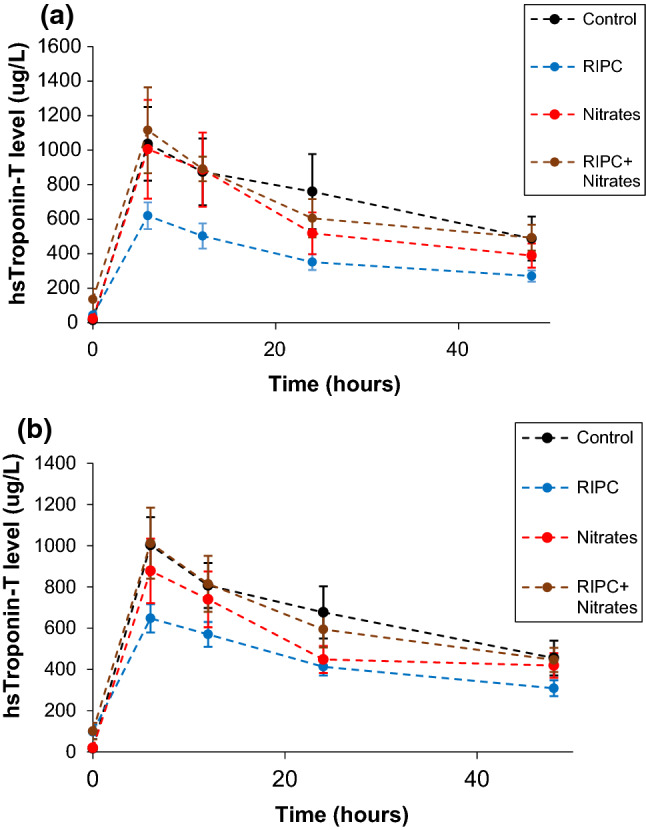
**a** Graph showing change in mean (SEM) hs-cTnT levels over 48 h in ERIC-GTN trial for dataset with completed hs-cTnT values [control (*n* = 20), RIPC (*n* = 26), GTN (*n* = 18), GT* n* + RIPC (*n* = 27)]. **b** Graph showing change in mean (SEM) hs-cTnT levels over 48 h in ERIC-GTN trial for dataset imputed for missing hs-cTnT values [control (*n* = 45) RIPC (*n* = 43), GTN (*n* = 48) GT* n* + RIPC (*n* = 49)]

189 patients were enrolled between January 2013 and February 2017, from which four patients were excluded from analysis due to missing data on treatment assignment (see consort diagram in Fig. 1). Detailed data for patients who were screened but excluded were not available. Patient recruitment into the ERIC-GTN study was prematurely terminated due to the results of a post-hoc analysis of the ERICCA study which found increased all-cause mortality at 12 months for RIPC used in combination with nitrates, when compared to control. 185 patients were randomly allocated to control (*n* = 45), RIPC alone (*n* = 43), nitrates alone (*n* = 48), and RIPC + Nitrates (*n* = 49) treatment groups. One patient from the RIPC group crossed over to the RIPC + GTN group as IV GTN was clinically indicated in that patient. There were no dropouts, and all patients completed the full protocol. The four treatment groups were well-balanced for baseline characteristics (Table 1), except that the control group had a lower prevalence of smokers, and the RIPC group had a higher prevalence of prior stroke. The number of patients who received propofol as part of the induction regime was 153/177 (86.4%), while the number of patients who received propofol as a maintenance infusion was 170/177 (96.0%). Figure 2 shows the graphs of post-operative 48-h AUC hs-cTnT for the four treatment groups in the complete hs-cTnT and imputed datasets and the data are tabulated in Supplementary Table 1.

**Table 1:** Baseline patient characteristics for ERIC-GTN study

Characteristics	Control *n* = 45	RIPC alone *n* = 43	Nitrates alone *n* = 47	RIPC + Nitrates *n* = 49
Age (years), mean (SEM)	68.5 (1.8)	66 (1.7)	66.8 (1.8)	67.9 (1.7)
Male gender, *n* (%)	33 (73)	34 (79)	40 (85)	37 (77)
Smoker, *n* (%)	22 (55)	27(67)	32 (74)	27(59)
BMI, mean (SEM)	29.2 (0.8)	28.3 (0.7)	29.5 (0.8)	28.5 (0.7)
LVEF (%), mean (SEM)	58.6 (1.3)	57.6 (1.9)	58.1 (1.3)	54.5 (1.6)
NYHA class, *n* (%)				
I	7 (20.6)	8 (23.5)	7 (24.1)	6 (21.5)
II	17 (50.0)	14 (41.2)	12 (41.4)	17 (51.5)
III	9 (26.5)	11 (32.3)	9 (31.0)	10 (30.3)
IV	1 (2.94)	1 (2.94)	1 (3.45)	0 (0)
Prior diagnoses, *n* (%)				
Diabetes mellitus	11 (24)	13 (30)	10 (21)	16 (33)
Hyperlipidaemia	21 (46)	27 (62)	27 (59)	21 (43)
Hypertension	27 (60)	30 (69)	30 (63)	29 (60)
MI	11 (24)	10 (23)	8 (17)	17 (35)
TIA/Stroke	2 (4)	6 (13)	2 (4)	3 (6)
*Medication at time of randomisation*				
Nitrates, *n* (%)	12 (26)	11 (25)	15 (31)	13 (27)
Beta-blocker, *n* (%)	29 (64.4)	30 (69.8)	26 (55.3)	33 (68.8)

*RIPC* remote ischaemic preconditioning; *SEM*, standard error of mean; *BMI*, body mass index; *LVEF*, left ventricular ejection fracture; *NYHA*, New York Heart Association; *TIA*, transient ischaemic attack

The pre-specified complete case analysis found that there was a significant interaction between RIPC and Nitrates with higher AUC hs-cTnT when the treatments were used together (interaction ratio of AUC 1.873, 95% CI 1.026–3.418, *p* = 0.041). Patient characteristics were similar between those included in the complete case analysis and those who were excluded. Table 2 shows pairwise comparisons of the primary endpoint between the four treatment groups. RIPC significantly reduced the primary endpoint of AUC hs-cTnT by 37.1%, when compared to control (ratio of AUC 0.629, 95% CI 0.413–0.957, *p* = 0.031). However, the cardioprotective effect of RIPC was completely abrogated in the presence of IV GTN (ratio of AUC 0.956, 95% CI 0.630–1.450, *p* = 0.829). GTN did not significantly reduce AUC hs-cTnT, when compared to control (ratio of AUC 0.812, 95% CI 0.513–1.285, *p* = 0.369). For the imputed dataset, pairwise comparisons were not statistically different, as well as the RIPC-Nitrates interaction coefficient (ratio of AUC 1.299, 95% CI 0.839–2.011, *p* = 0.239).

**Table 2:** Pairwise comparisons of logarithmic 48-h AUC hs-cTnT for ERIC-GTN study

	Ratio of AUC geometric mean	Unadjusted 95% Confidence interval		*p* value
*Complete hs-cTnT dataset (n* = 91)*				
RIPC alone vs. Control	0.629	0.413	0.957	0.031*
Nitrates alone vs. Control	0.812	0.513	1.285	0.369
RIPC + Nitrates vs. Control	0.956	0.630	1.450	0.829
Nitrates alone vs. RIPC alone	1.291	0.837	1.991	0.245
RIPC + Nitrates vs. RIPC alone	1.520	1.031	2.242	0.035*
RIPC + Nitrates vs. Nitrates alone	1.177	0.766	1.810	0.453
*Multiple imputation dataset (n* = 185)**				
RIPC alone vs. Control	0.958	0.679	1.353	0.807
Nitrates alone vs. Control	0.769	0.549	1.078	0.126
RIPC + Nitrates vs. Control	0.996	0.717	1.383	0.981
Nitrates alone vs. RIPC alone	1.246	0.871	1.782	0.226
RIPC + Nitrates vs. RIPC alone	1.295	0.914	1.834	0.144
RIPC + Nitrates vs. Nitrates alone	1.039	0.735	1.470	0.826

Coefficients and confidence intervals have been exponentiated for convenience

*Coefficient for interaction term was statistically significant (*F*-test, *p* = 0.041)

**Coefficient for interaction term was not statistically significant (*F*-test, *p* = 0.239)

### ERICCA post-hoc analysis

**Fig. 3:**
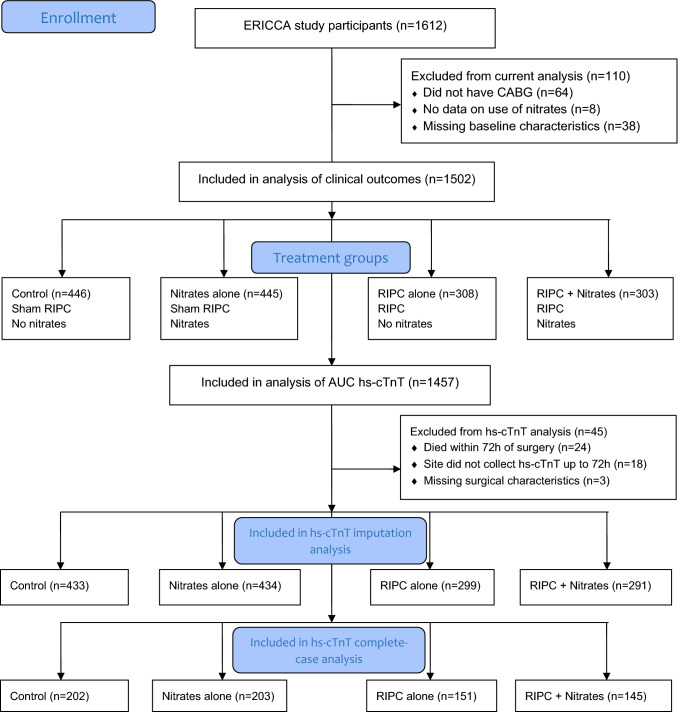
Patient flow diagram for ERICCA post-hoc analysis

The analysis included 1502 patients, divided into 446 (30%) controls who had neither RIPC nor nitrates (IV GTN or long-acting oral nitrates), 445 (30%) who had RIPC but no nitrates, 308 (21%) who had nitrates but not RIPC, and 303 (20%) who had both RIPC and nitrates (Fig. 3). There was a good balance in patient characteristics between those who received RIPC and those who received sham RIPC. However, those who received nitrates had a higher BMI and were more likely to have been diagnosed with diabetes (and be treated with medications for diabetes), have a previous MI; have higher CCS angina class, and to have a family history of ischaemic heart disease in comparison to those who did not receive nitrates (Table 3; Supplementary Tables 2 and 3). There were large differences between the study sites in nitrate use, with some sites having few patients treated with nitrates and others where the majority were treated with IV GTN or long-lasting oral nitrates at baseline.

**Table 3:** Baseline patient characteristics for ERICCA post-hoc analysis by RIPC and nitrates (either intraoperative IV GTN or long-lasting oral nitrates)

	Control		RIPC alone		Nitrates alone		RIPC + Nitrates	
	*N* = 446		*N* = 445		*N*= 308		*N*= 303	
*Age (years)*								
Mean (SEM)	76.2	(0.3)	76.4	(0.3)	76.2	(0.4)	75.7	(0.3)
Range (min, max)		[20, 92]		[52, 93]		[44, 93]		[50, 87]
Sex—*N* (%) Female	118	(26.5)	129	(29.0)	89	(28.9)	86	(28.4)
*BMI (kg/m*^*2*^*)*								
Mean (SEM)	27.5	(0.2)	27.2	(0.2)	27.6	(0.2)	28.3	(0.2)
Range (min, max)		[16, 45]		[17, 45]		[18, 39]		[19, 45]
*Smoking baseline—N (%)*								
Never	151	(33.9)	170	(38.2)	107	(34.7)	101	(33.3)
Ex	266	(59.6)	251	(56.4)	180	(58.4)	186	(61.4)
Current	29	(6.5)	24	(5.4)	21	(6.8)	16	(5.3)
*Past medical history—N (%)*								
Diabetes Mellitus	115	(25.8)	104	(23.4)	84	(27.3)	87	(28.7)
Hypercholesterolaemia	308	(69.1)	316	(71.0)	213	(69.2)	221	(72.9)
Hypertension	331	(74.2)	344	(77.3)	232	(75.3)	227	(74.9)
MI	159	(35.7)	167	(37.5)	136	(44.2)	145	(47.9)
PCI	61	(13.7)	63	(14.2)	53	(17.2)	45	(14.9)
CABG	7	(1.6)	13	(2.9)	10	(3.2)	11	(3.6)
Stroke	51	(11.4)	58	(13.0)	34	(11.0)	36	(11.9)
Atrial fibrillation	76	(17.0)	60	(13.5)	54	(17.5)	49	(16.2)
Peripheral arterial disease	29	(6.5)	32	(7.2)	31	(10.1)	26	(8.6)
Family history IHD	163	(36.5)	158	(35.5)	140	(45.5)	140	(46.2)
*Euroscore*								
Mean (SEM)	6.7	(0.1)	6.6	(0.1)	6.6	(0.1)	6.5	(0.1)
Range (min, max)		[5, 13]		[5, 15]		[5, 16]		[5, 17]
Valve repair/replace—*N* (%)	284	(54.0)	250	(48.2)	109	(47.8)	108	(47.2)
*Cross-clamp time (minutes)*								
Mean (SEM)	77.5	(1.9)	77.1	(1.9)	72.7	(2.4)	70.5	(2.1)
Range (min, max)		[0, 292]		[0, 314]		[0, 290]		[0, 324]
*Cardiopulmonary bypass time (minutes)*								
Mean (SEM)	114.3	(2.4)	114.7	(2.5)	109.4	(2.9)	107.3	(2.8)
Range (min, max)		[0, 382]		[0, 585]		[0, 422]		[0, 506]
Propofol—*N* (%)	395	(91.2)	403	(92.6)	285	(94.7)	290	(95.7)
Volatile anesthetics—*N* (%)	171	(39.5)	179	(41.1)	147	(48.8)	141	(46.5)

*N*, number; %, percentage; *SEM*, standard error of mean; *BMI*, body mass index; *MI*, myocardial infarction; *PCI*, Percutaneous coronary intervention; *CABG*, coronary artery bypass; *PAD*, peripheral artery disease; *IHD*, ischaemic heart disease

**Fig. 4:**
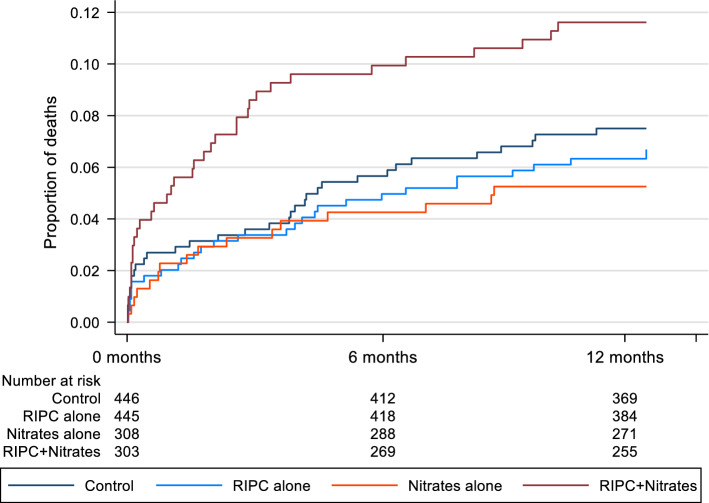
Kaplan–Meier estimate of cumulative incidence of all-cause mortality up to 12 months, by RIPC and nitrates (either intraoperative IV GTN or long-lasting oral nitrates) for ERICCA post-hoc analysis

For the primary analysis endpoint of 12-month all-cause mortality, the effects of RIPC and nitrates are shown in Tables 4 and 5. There was evidence of an interaction between RIPC and nitrates (*p* = 0.008, adjusted analysis), such that those who received both RIPC and nitrates (with either IV GTN or long-acting oral nitrates) had higher all-cause mortality at 12 months than would be expected from the effects of each treatment given in isolation (Tables 4, 5; Fig. 4). Mortality did not significantly differ between the control group and those who received nitrates alone (no RIPC) or RIPC alone (without nitrates) but was 2.16 times higher compared to control in those who received RIPC in combination with nitrates (HR = 2.16, 95% CI 1.22–3.82, *p* = 0.008, adjusted analysis Table 5; Fig. 4). However, when considering IV GTN only irrespective of use of long-term use of oral nitrates, the interaction with RIPC was weaker, and did not reach statistical significance (*p* = 0.135, Supplementary Table 4 and Fig. 2).

**Table 4:** Clinical outcomes by treatment with RIPC and nitrates (either intraoperative IV GTN or long-lasting oral nitrates) for ERICCA post-hoc analysis

	Control		RIPC alone		Nitrates alone		RIPC + Nitrates	
	*N* = 446		*N* = 445		*N* = 308		*N* = 303	
All-cause mortality—*N* (%)	33	(7.4)	29	(6.5)	16	(5.2)	35	(11.6)
Cardiovascular mortality—*N* (%)	20	(4.5)	20	(4.5)	7	(2.3)	22	(7.3)
Peri-operative MI—*N* (%)	127	(28.5)	98	(22.0)	55	(17.9)	65	(21.5)
72-h AUC hs-cTnT—mean (SEM)								
Complete case data (*N* = 701)	51,569	(2313)	44,070	(1739)	42,158	(1979)	38,540	(1724)
Multiple imputation data (*N* = 1460)^a^	50,106	(2205)	46,715	(2043)	42,619	(1989)	41,916	(1928)

*N*, number; %, percent; *SEM*, standard error of mean

^a^42 patients excluded from multiple imputation, reasons for exclusions were: 24 died before 72 h; 18 patients at sites that did not collect hs-cTnT up to 72 h

**Table 5:** Effect of RIPC and nitrates (either intraoperative IV GTN or long-lasting oral nitrates) on all-cause mortality up to 12 months following surgery for ERICCA post-hoc analysis

	Unadjusted^a^				Adjusted^b^			
	HR	95% CI		*p* value	HR	95% CI		*P* value
Interaction nitrates*RIPC	2.78	1.27	6.09	0.010	2.88	1.31	6.32	0.008
*Effect of RIPC in strata*								
No nitrates	0.85	0.52	1.40	0.529	0.91	0.55	1.51	0.714
Nitrates	2.37	1.30	4.31	0.005	2.62	1.43	4.79	0.002
**Comparison of each combination**								
*Control (sham RIPC without nitrates) (ref)*								
RIPC alone	0.85	0.52	1.40	0.529	0.91	0.55	1.51	0.714
Nitrates alone	0.79	0.41	1.53	0.482	0.83	0.43	1.60	0.572
RIPC + Nitrates	1.87	1.07	3.28	0.029	2.16	1.22	3.82	0.008

*HR*, hazard ratio; *CI*, confidence interval

^a^Cox Proportional hazards model with stratification by study site

^b^Cox Proportional hazards model with stratification by study site and adjustment for EuroSCORE, body mass index (BMI), previous myocardial infarction, and diabetes

**Fig. 5:**
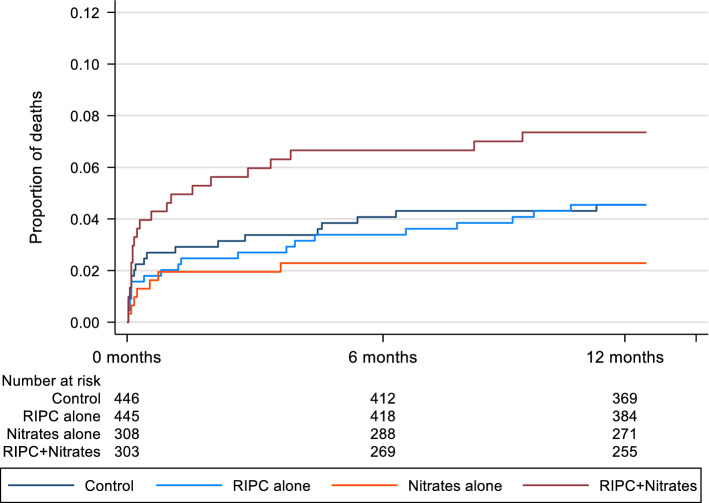
Kaplan–Meier estimate of cumulative incidence of cardiovascular mortality, by RIPC and nitrates (either intraoperative IV GTN or long-lasting oral nitrates) for ERICCA post-hoc analysis

There was evidence of an interaction between RIPC and nitrates for 12-month cardiovascular mortality (*p* = 0.024, adjusted analysis; Tables 4, 6; Fig. 5). Cardiovascular mortality was 2.24 times higher in those who received RIPC in combination with nitrates compared to control (adjusted HR = 2.24, 95% CI 1.09–4.58, *p* = 0.028: Table 6; Fig. 5). Cardiovascular mortality was similar to the control group for those who had RIPC alone (without nitrates). Although there was a trend towards lower cardiovascular mortality for nitrates alone (no RIPC) compared to controls this did not reach statistical significance (Tables 4, 6; Fig. 5).

**Table 6:** Effect of RIPC and nitrates (either intraoperative IV GTN or long-lasting oral nitrates) on cardiovascular mortality up to 12 months following surgery for ERICCA post-hoc analysis

	Unadjusted^a^				Adjusted^b^			
	HR	95% CI		*p* value	HR	95% CI		*p* value
Interaction Nitrates * RIPC	3.45	1.19	9.97	0.022	3.40	1.17	9.86	0.024
*Effect of RIPC in strata*								
No nitrates	0.98	0.53	1.82	0.947	1.07	0.57	2.00	0.830
Nitrates	3.38	1.43	7.96	0.005	3.64	1.54	8.61	0.003
**Comparison of each combination**								
*Control (sham RIPC without nitrates) (ref)*								
RIPC alone	0.98	0.53	1.82	0.947	1.07	0.57	2.00	0.830
Nitrates alone	0.57	0.23	1.44	0.236	0.61	0.24	1.56	0.306
RIPC + Nitrates	1.93	0.95	3.93	0.070	2.24	1.09	4.58	0.028

*HR*, hazard ratio; *CI*, confidence interval

^a^Cox Proportional hazards model with stratification by study site

^b^Cox Proportional hazards model with stratification by study site and adjustment for EuroSCORE, body mass index (BMI), previous myocardial infarction, and diabetes

For perioperative MI, there was evidence of an interaction between RIPC and nitrates (*p* = 0.025, adjusted analysis; Tables 4, 7). The risk of perioperative MI was significantly lower for those who received RIPC alone (no nitrates) compared to control (adjusted odds ratio (OR) = 0.69; 95% CI 0.50–0.95; *p* = 0.025; Tables 4, 7) and there was weak evidence for a reduction for those who received nitrates alone (no RIPC) compared to control (adjusted odds ratio (OR) = 0.69; 95% CI 0.46–1.03; *p* = 0.069; Tables 4, 7). The reduction in risk compared to control for those who received both RIPC and nitrates was smaller and not statistically significant [adjusted odds ratio (OR) = 0.87; 95% CI 0.59–1.28; *p* = 0.481; Tables 4, 7].

**Table 7:** Effect of RIPC and nitrates (either intraoperative IV GTN or long-lasting oral nitrates) on perioperative myocardial infarction for ERICCA post-hoc analysis

	Unadjusted				Adjusted^a^			
	OR	95% CI		*p* value	OR	95% CI		*p* value
Interaction nitrates * RIPC	1.78	1.06	3.00	0.029	1.82	1.08	3.06	0.025
*Effect of RIPC in strata*								
No nitrates	0.70	0.51	0.96	0.025	0.69	0.50	0.95	0.025
Nitrates	1.24	0.83	1.87	0.299	1.26	0.84	1.89	0.271
Comparison of each combination								
*Control (sham RIPC without nitrates) (ref)*								
RIPC alone	0.70	0.51	0.96	0.025	0.69	0.50	0.95	0.025
Nitrates alone	0.68	0.46	1.01	0.056	0.69	0.46	1.03	0.069
RIPC + Nitrates	0.84	0.57	1.24	0.386	0.87	0.59	1.28	0.481

*OR*, odds ratio; *CI*, confidence interval

^a^Adjusted for EuroSCORE, body mass index (BMI), previous myocardial infarction, and diabetes

Regarding PMI, only 701 (47%) had a complete set of hs-cTnT data, and so the analysis was performed on the imputed dataset (Tables 4, 7). There was little evidence of an interaction between RIPC and nitrates (*p* = 0.295, adjusted analysis) or for difference in PMI by treatment with RIPC or nitrates or their combination (Table 8).

**Table 8:** Effect of RIPC and nitrates (either intraoperative IV GTN or long-lasting oral nitrates) on peri-operative myocardial injury for ERICCA post-hoc analysis

	Unadjusted^#^				Adjusted^#^*			
	Ratio	95% CI		*p* value	Ratio	95% CI		*p* value
Interaction nitrates * RIPC	1.07	0.92	1.25	0.358	1.09	0.94	1.26	0.276
*Effect of RIPC in strata*								
No nitrates	0.93	0.84	1.04	0.190	0.93	0.84	1.03	0.185
Nitrates	1.00	0.89	1.13	0.983	1.01	0.90	1.14	0.820
**Comparison of each combination**								
*Control (sham RIPC without nitrates) (ref)*								
RIPC alone	0.93	0.84	1.04	0.190	0.93	0.84	1.03	0.185
Nitrates alone	0.93	0.83	1.05	0.251	0.95	0.85	1.06	0.361
RIPC + Nitrates	0.94	0.83	1.06	0.292	0.96	0.85	1.09	0.528

CI, confidence interval. Ratio, ratio of geometric mean 72-h AUC hs-TnT

*Adjusted for EuroSCORE, body mass index (BMI), previous myocardial infarction, and diabetes

^#^Results using multiple imputation dataset

In the secondary analysis looking at IV GTN without use of long-term use of oral nitrates, the interactions with RIPC were smaller and did not reach statistical significance. In addition, the reduction in risk of perioperative MI for RIPC alone (no nitrates) compared to control was slightly smaller and did not reach formal statistical significance at the 5% level (Supplementary Tables 4–7 and Figs. 1, 2). Interestingly, in the secondary analysis evaluating the effect of long-term use of oral nitrates without use of IV GTN, the interactions with RIPC were stronger with the greatest impact observed in all-cause mortality at 12 months (Supplementary Tables 8–11 and Figs. 3, 4).

## Discussion

The findings from the ERIC-GTN study and the post-hoc analysis of the ERICCA study suggest that there was a negative interaction between RIPC and nitrates, with the presence of concomitant nitrates abrogating RIPC-induced cardioprotection and increasing 12-months’ all-cause and cardiovascular mortality in patients undergoing CABG ± valve surgery.

In the ERIC-GTN study, we found that RIPC in the absence of IV GTN reduced PMI (quantified by 48 h-AUC-hs-cTnT), and the findings from our post-hoc analysis of the ERICCA study suggested that RIPC in the absence of nitrates also reduced the risk of peri-operative MI. These findings confirm the cardioprotective effects of RIPC in patients undergoing CABG ± valve surgery observed in several previous studies [11, 34, 35], some of which also showed improved short-term [6] and long-term outcomes [35]. However, these findings appear to be in conflict with the ERICCA [12] and RIPHeart [27] studies which failed to demonstrate a reduction in the extent of PMI. The difference may relate to the stronger RIPC stimulus used in the ERIC-GTN trial in which limb RIPC was simultaneously applied to both the arm and leg, whereas in the ERICCA and RIPHeart trials, limb RIPC was only applied to the arm. This concept has been supported by one clinical study showing greater endothelial protection with RIPC applied to the leg when compared to the arm [26], although a prior experimental study in mice failed to show superior cardioprotection with bilateral hindlimb ischaemia when compared to single hindlimb ischaemia [19]. Interestingly, the cardioprotective effects of RIPC observed in the ERIC-GTN and ERICCA studies in the absence of nitrates, were present despite the majority of patients being administered propofol anaesthesia, the latter of which has been reported in experimental [2, 4, 7, 8, 41] and clinical studies [1, 23–25] to abrogate RIPC-induced cardioprotection. This finding suggests, that in our studies, nitrates rather than propofol may have confounded RIPC-induced cardioprotection.

The effects of RIPC alone on long-term outcomes in the absence of intraoperative GTN and/or long-term oral nitrates were less clear. RIPC alone had no significant effect on either all-cause or cardiovascular mortality, but this post-hoc analysis may have been underpowered to detect an effect in this subgroup. Whether the presence of intraoperative GTN and/or long-term oral nitrates contributed to the lack of cardioprotection observed in the RIPHeart study is not known. In the post-hoc ERICCA analysis, the negative interaction between RIPC and nitrates was most marked when both IV GTN and long-term oral nitrates were given, with the interaction no longer being significant when only IV GTN was considered. This finding may be because the comparator control group included patients on long-term oral nitrates. When considered individually, the negative interaction between RIPC and nitrates was strongest with long-term oral nitrates alone when compared to IV GTN alone.

Our findings suggest a negative interaction between RIPC and nitrates in patients undergoing CABG ± valve surgery, with the observed effects of RIPC reducing PMI in the ERIC-GTN study and reducing the risk of peri-operative MI in the post-hoc analysis of the ERICCA study, being abrogated by the presence of nitrates. This finding is in concordance with previous studies reporting that either acute of chronic treatment with NO-donors abolished both RIPC-induced cardioprotection in animal models of acute myocardial IRI [10, 33], and RIPC-induced vasculoprotection in human volunteers [10]. Furthermore, these findings support the post-hoc retrospective analysis showing that the cardioprotective effect of RIPC in reducing PMI was abolished in patients administered intraoperative IV GTN [6], although this negative interaction between IV GTN and RIPC was not demonstrated in a post-hoc analysis by Kleinbongard et al. [22] The reasons for the discordant findings are not clear but may relate to the small sample size and anaesthetics used in these studies, the study design (retrospective vs. prospective), and the duration of IV GTN administration. The mechanism through which nitrates block RIPC-induced cardioprotection is not known, but may relate to NO inhibiting afferent nerve conduction in the limb, required for the limb RIPC stimulus to mediate cardioprotection [33]. Of more concern however, was our finding that patients administered both RIPC and nitrates had significantly higher rates of all-cause and cardiovascular mortality according to the ERICCA post-hoc analysis, the mechanisms of which are unclear and need further investigation.

Even though the cardioprotective effects of nitrates are well-established [3], whether nitrates are cardioprotective in patients undergoing CABG ± valve surgery is not clear with mixed results from clinical studies [6] and meta-analyses [18], although prospective randomised controlled trials have been limited. Prior evidence that suggest a possible protective effect of GTN include a 2017 meta-analysis of secondary data from five small trials (total 180 patients) in patients undergoing cardiac surgery which showed a trend towards less PMI but it did not reach statistical significance [18]. A post-hoc retrospective analysis in CABG ± valve patients found that patients administered intraoperative IV GTN for clinical indications, administered after induction of anaesthesia and stopped when patient went onto cardiopulmonary bypass, had 39% less PMI (assessed by 72 h-AUC-hs-cTnT), when compared to control [6]. However, a post-hoc retrospective analysis of another CABG study failed to observe cardioprotection with intraoperative IV GTN for clinical indications, administered after induction of anaesthesia and stopped when patient went onto cardiopulmonary bypass [22]. In the ERIC-GTN study, we also failed to demonstrate cardioprotection with intraoperative IV GTN initiated at a dose of 2 mg/h after induction of anaesthesia and stopped when the patient came off cardiopulmonary bypass in patients undergoing CABG ± valve surgery, when compared to control. Potential reasons for this discrepancy include the small sample sizes, no standardised protocols for starting or titrating intraoperative IV GTN for clinical indications, and the post-hoc nature of the analyses with the related statistical limitations. Interestingly, in our post-hoc analysis of the ERICCA study, we observed a non-significant reduction in the risk of peri-operative MI and all-cause and cardiovascular mortality at 12 months in patients given IV GTN for clinical indications or previously on long-lasting oral nitrates, when compared to control. However, a suitably powered clinical prospective randomised controlled trial is needed to evaluate whether intraoperative IV GTN or prior oral nitrate therapy can reduce risk of peri-operative MI and improve clinical outcomes in patients undergoing cardiac bypass surgery.

There are several limitations to the ERIC-GTN study. Due to the premature termination of patient recruitment, the study was underpowered. The optimal RIPC protocol is yet to be characterised in terms of dosing (number and length of cycles) and timing (before CPB), and it is possible that the true effect size of RIPC was underestimated. The primary endpoint of the study was PMI and not the clinically relevant, type 5 MI, as defined by the Universal Definition of Myocardial Infarction (UDMI) [38]. Finally, due to missing hs-cTnT data, we had to perform two different analyses, a complete hs-cTnT dataset analysis which showed RIPC reducing PMI when compared to control, and an imputed hs-cTnT dataset analysis which only showed a non-significant reduction in PMI with RIPC, when compared to control.

In conclusion, in our prospective ERIC-GTN randomised controlled trial, we showed that RIPC reduced the extent of PMI in patients undergoing cardiac bypass surgery, but this cardioprotective effect was attenuated in the presence of intraoperative IV GTN, suggesting a negative interaction between RIPC and nitrates with respect to cardioprotection. Furthermore, in a post-hoc analysis of the ERICCA study, we found that the effect of RIPC in reducing the risk of peri-operative MI was abrogated in patients given nitrates, and of more concern we showed higher rates of all-cause and cardiovascular mortality in patients given both RIPC and nitrates, confirming the negative interaction between the two on clinical outcomes in patients undergoing cardiac bypass surgery. These findings may, in part, explain the neutral results of RIPC on clinical outcomes in the ERICCA and RIPHeart trials. This interaction may have potential implications on the application of RIPC in other clinical settings such as acute myocardial infarction, where patients frequently receive sublingual or intravenous GTN or are already on long-term oral nitrate therapy, and may therefore explain, in part, the neutral results in the CONDI-2/ERIC-PPCI, which failed to demonstrate an improvement of clinical outcomes with RIPC in acute ST-segment elevation myocardial infarction patients treated by PPCI [13]. As such, the negative interaction between nitrates and RIPC should be considered when designing future clinical studies evaluating the cardioprotective effects of RIPC.

## Supplementary Information

Below is the link to the electronic supplementary material.

Supplementary file1 (DOCX 72 kb)

## References

[1] Abel F, Murke F, Gaida M, Garnier N, Ochsenfarth C, Theiss C, Thielmann M, Kleinbongard P, Giebel B, Peters J, Frey UH (2020). Extracellular vesicles isolated from patients undergoing remote ischemic preconditioning decrease hypoxia-evoked apoptosis of cardiomyoblasts after isoflurane but not propofol exposure. PLoS ONE.

[2] Behmenburg F, van CP, Bunte S, Brandenburger T, Heinen A, Hollmann MW, Huhn R, (2018). Impact of anesthetic regimen on remote ischemic preconditioning in the rat heart in vivo. Anesth Analg.

[3] Bice JS, Jones BR, Chamberlain GR, Baxter GF (2016). Nitric oxide treatments as adjuncts to reperfusion in acute myocardial infarction: a systematic review of experimental and clinical studies. Basic Res Cardiol.

[4] Bunte S, Behmenburg F, Eckelskemper F, Mohr F, Stroethoff M, Raupach A, Heinen A, Hollmann MW, Huhn R (2019). Cardioprotection by humoral factors released after remote ischemic preconditioning depends on anesthetic regimen. Crit Care Med.

[5] Candilio L, Hausenloy D (2017) Is there a role for ischaemic conditioning in cardiac surgery? F1000Res 6:563. 10.12688/f1000research.10963.1 10.12688/f1000research.10963.1 PMC5405791 28503301

[6] Candilio L, Malik A, Ariti C, Barnard M, Di SC, Lawrence D, Hayward M, Yap J, Roberts N, Sheikh A, Kolvekar S, Hausenloy DJ, Yellon DM (2015). Effect of remote ischaemic preconditioning on clinical outcomes in patients undergoing cardiac bypass surgery: a randomised controlled clinical trial. Heart.

[7] Chen K, Yu J, Wang Q, Wu L, Liu X, Wong GTC, Lu Y (2020). The timing of propofol administration affects the effectiveness of remote ischemic preconditioning induced cardioprotection in rats. J Cell Biochem.

[8] Cho YJ, Nam K, Kim TK, Choi SW, Kim SJ, Hausenloy DJ, Jeon Y (2019). Sevoflurane, propofol and carvedilol block myocardial protection by limb remote ischemic preconditioning. Int J Mol Sci..

[9] Hamarneh A, Sivaraman V, Bulluck H, Shanahan H, Kyle B, Ramlall M, Chung R, Jarvis C, Xenou M, Ariti C, Cordery R, Yellon DM, Hausenloy DJ (2015). The effect of remote ischemic conditioning and glyceryl trinitrate on perioperative myocardial injury in cardiac bypass surgery patients: rationale and design of the ERIC-GTN study. Clin Cardiol.

[10] Hauerslev M, Mork SR, Pryds K, Contractor H, Hansen J, Jespersen NR, Johnsen J, Heusch G, Kleinbongard P, Kharbanda R, Botker HE, Schmidt MR (2018). Influence of long-term treatment with glyceryl trinitrate on remote ischemic conditioning. Am J Physiol Heart Circ Physiol.

[11] Hausenloy DJ, Mwamure PK, Venugopal V, Harris J, Barnard M, Grundy E, Ashley E, Vichare S, Di Salvo C, Kolvekar S, Hayward M, Keogh B, MacAllister RJ, Yellon DM (2007). Effect of remote ischaemic preconditioning on myocardial injury in patients undergoing coronary artery bypass graft surgery: a randomised controlled trial. Lancet.

[12] Hausenloy DJ, Candilio L, Evans R, Ariti C, Jenkins DP, Kolvekar S, Knight R, Kunst G, Laing C, Nicholas J, Pepper J, Robertson S, Xenou M, Clayton T, Yellon DM (2015). Remote ischemic preconditioning and outcomes of cardiac surgery. N Engl J Med.

[13] Hausenloy DJ, Kharbanda RK, Moller UK, Ramlall M, Aaroe J, Butler R, Bulluck H, Clayton T, Dana A, Dodd M, Engstrom T, Evans R, Lassen JF, Christensen EF, Garcia-Ruiz JM, Gorog DA, Hjort J, Houghton RF, Ibanez B, Knight R, Lippert FK, Lonborg JT, Maeng M, Milasinovic D, More R, Nicholas JM, Jensen LO, Perkins A, Radovanovic N, Rakhit RD, Ravkilde J, Ryding AD, Schmidt MR, Riddervold IS, Sorensen HT, Stankovic G, Varma M, Webb I, Terkelsen CJ, Greenwood JP, Yellon DM, Botker HE (2019). Effect of remote ischaemic conditioning on clinical outcomes in patients with acute myocardial infarction (CONDI-2/ERIC-PPCI): a single-blind randomised controlled trial. Lancet.

[14] Heusch G (2001). Nitroglycerin and delayed preconditioning in humans: yet another new mechanism for an old drug?. Circulation.

[15] Heusch G (2017). Remote ischemic conditioning in cardiovascular surgery. J Cardiovasc Pharmacol Ther.

[16] Ho AFW, Chong J, Ong MEH, Hausenloy DJ (2020). Remote ischemic conditioning in emergency medicine-clinical frontiers and research opportunities. Shock.

[17] Hong DM, Lee EH, Kim HJ, Min JJ, Chin JH, Choi DK, Bahk JH, Sim JY, Choi IC, Jeon Y (2014). Does remote ischaemic preconditioning with postconditioning improve clinical outcomes of patients undergoing cardiac surgery? Remote Ischaemic Preconditioning with Postconditioning Outcome Trial. Eur Heart J.

[18] Hoshijima H, Denawa Y, Mihara T, Takeuchi R, Kuratani N, Mieda T, Iwase Y, Shiga T, Wajima Z, Nagasaka H (2017). Efficacy of prophylactic doses of intravenous nitroglycerin in preventing myocardial ischemia under general anesthesia: a systematic review and meta-analysis with trial sequential analysis. J Clin Anesth.

[19] Johnsen J, Pryds K, Salman R, Lofgren B, Kristiansen SB, Botker HE (2016). The remote ischemic preconditioning algorithm: effect of number of cycles, cycle duration and effector organ mass on efficacy of protection. Basic Res Cardiol.

[20] Karuppasamy P, Chaubey S, Dew T, Musto R, Sherwood R, Desai J, John L, Shah AM, Marber MS, Kunst G (2011). Remote intermittent ischemia before coronary artery bypass graft surgery: a strategy to reduce injury and inflammation?. Basic Res Cardiol.

[21] Kharbanda RK, Mortensen UM, White PA, Kristiansen SB, Schmidt MR, Hoschtitzky JA, Vogel M, Sorensen K, Redington AN, MacAllister R (2002). Transient limb ischemia induces remote ischemic preconditioning in vivo. Circulation.

[22] Kleinbongard P, Thielmann M, Jakob H, Peters J, Heusch G, Kottenberg E (2013). Nitroglycerin does not interfere with protection by remote ischemic preconditioning in patients with surgical coronary revascularization under isoflurane anesthesia. Cardiovasc Drugs Ther.

[23] Kleinbongard P, Botker HE, Ovize M, Hausenloy DJ, Heusch G (2020). Co-morbidities and co-medications as confounders of cardioprotection—does it matter in the clinical setting?. Br J Pharmacol.

[24] Kottenberg E, Thielmann M, Bergmann L, Heine T, Jakob H, Heusch G, Peters J (2012). Protection by remote ischemic preconditioning during coronary artery bypass graft surgery with isoflurane but not propofol—a clinical trial. Acta Anaesthesiol Scand.

[25] Kottenberg E, Musiolik J, Thielmann M, Jakob H, Peters J, Heusch G (2014). Interference of propofol with signal transducer and activator of transcription 5 activation and cardioprotection by remote ischemic preconditioning during coronary artery bypass grafting. J Thorac Cardiovasc Surg.

[26] Loukogeorgakis SP, Williams R, Panagiotidou AT, Kolvekar SK, Donald A, Cole TJ, Yellon DM, Deanfield JE, MacAllister RJ (2007). Transient limb ischemia induces remote preconditioning and remote postconditioning in humans by a K(ATP)-channel dependent mechanism. Circulation.

[27] Meybohm P, Bein B, Brosteanu O, Cremer J, Gruenewald M, Stoppe C, Coburn M, Schaelte G, Boning A, Niemann B, Roesner J, Kletzin F, Strouhal U, Reyher C, Laufenberg-Feldmann R, Ferner M, Brandes IF, Bauer M, Stehr SN, Kortgen A, Wittmann M, Baumgarten G, Meyer-Treschan T, Kienbaum P, Heringlake M, Schon J, Sander M, Treskatsch S, Smul T, Wolwender E, Schilling T, Fuernau G, Hasenclever D, Zacharowski K (2015). A multicenter trial of remote ischemic preconditioning for heart surgery. N Engl J Med.

[28] Nielsen S, Bjorck L, Jeppsson A, Giang KW, Falk K, Maatta S, Sandstrom TZ, Rosengren A (2017). Trends in mortality risks among 94,328 patients surviving 30 days after a first isolated coronary artery bypass graft procedure from 1987 to 2006: a population-based study. Int J Cardiol.

[29] Przyklenk K, Bauer B, Ovize M, Kloner RA, Whittaker P (1993). Regional ischemic 'preconditioning' protects remote virgin myocardium from subsequent sustained coronary occlusion. Circulation.

[30] Rahman IA, Mascaro JG, Steeds RP, Frenneaux MP, Nightingale P, Gosling P, Townsend P, Townend JN, Green D, Bonser RS (2010). Remote ischemic preconditioning in human coronary artery bypass surgery: from promise to disappointment?. Circulation.

[31] Rubin DB (2004). Multiple imputation for nonresponse in surveys.

[32] Sivaraman V, Pickard JM, Hausenloy DJ (2015). Remote ischaemic conditioning: cardiac protection from afar. Anaesthesia.

[33] Steensrud T, Li J, Dai X, Manlhiot C, Kharbanda RK, Tropak M, Redington A (2010). Pretreatment with the nitric oxide donor SNAP or nerve transection blocks humoral preconditioning by remote limb ischemia or intra-arterial adenosine. Am J Physiol Heart Circ Physiol.

[34] Thielmann M, Kottenberg E, Boengler K, Raffelsieper C, Neuhaeuser M, Peters J, Jakob H, Heusch G (2010). Remote ischemic preconditioning reduces myocardial injury after coronary artery bypass surgery with crystalloid cardioplegic arrest. Basic Res Cardiol.

[35] Thielmann M, Kottenberg E, Kleinbongard P, Wendt D, Gedik N, Pasa S, Price V, Tsagakis K, Neuhauser M, Peters J, Jakob H, Heusch G (2013). Cardioprotective and prognostic effects of remote ischaemic preconditioning in patients undergoing coronary artery bypass surgery: a single-centre randomised, double-blind, controlled trial. Lancet.

[36] Thielmann M, Sharma V, Al-Attar N, Bulluck H, Bisleri G, Jh BJ, Czerny M, Ferdinandy P, Frey UH, Heusch G, Holfeld J, Kleinbongard P, Kunst G, Lang I, Lentini S, Madonna R, Meybohm P, Muneretto C, Obadia JF, Perrino C, Prunier F, Sluijter JPG, van Laake LW, Sousa-Uva M, Hausenloy DJ (2017). ESC joint working groups on cardiovascular surgery and the cellular biology of the heart position paper: perioperative myocardial injury and infarction in patients undergoing coronary artery bypass graft surgery. Eur Heart J.

[37] Thorsteinsson K, Fonager K, Merie C, Gislason G, Kober L, Torp-Pedersen C, Mortensen RN, Andreasen JJ (2016). Age-dependent trends in postoperative mortality and preoperative comorbidity in isolated coronary artery bypass surgery: a nationwide study. Eur J Cardiothorac Surg.

[38] Thygesen K, Alpert JS, Jaffe AS, Chaitman BR, Bax JJ, Morrow DA, White HD (2019). Fourth universal definition of myocardial infarction. Eur Heart J.

[39] White IR, Royston P, Wood AM (2011). Multiple imputation using chained equations: issues and guidance for practice. Stat Med.

[40] Yellon DM, He Z, Khambata R, Ahluwalia A, Davidson SM (2018). The GTN patch: a simple and effective new approach to cardioprotection?. Basic Res Cardiol.

[41] Yu J, Chen K, Wu L, Liu X, Lu Y (2019). Anesthetic propofol blunts remote preconditioning of trauma-induced cardioprotection via the TRPV1 receptor. Biomed Pharmacother.

