# Supplementary material for: Negative interaction between nitrates and remote ischemic preconditioning in patients undergoing cardiac surgery: the ERIC-GTN and ERICCA studies
Source: Basic Res Cardiol. 2022 Jun 21;117(1):31. doi: 10.1007/s00395-022-00938-3 (PMC9213287; doi:10.1007/s00395-022-00938-3)
Supplement: Supplementary file 1 — Supplementary file1 (DOCX 72 kb) [file 395_2022_938_MOESM1_ESM.docx]

**SUPPLEMENTARY TABLES**

**Supplementary Table 1. 48-hour AUC hs-cTnT values for patients in the ERIC-GTN trial for the complete and imputed hs-cTnT datasets.**

| **Group** | **N number** | **Mean 48-hour AUC hs-cTnT (ng/L)** | **SEM** |
| --- | --- | --- | --- |
| **Complete hs-cTnT dataset** | | | |
| **Control** | 20 | 33775 | 8319 |
| **RIPC** | 26 | 17946 | 2228 |
| **Nitrates** | 18 | 28085 | 6910 |
| **RIPC+Nitrates** | 27 | 31934 | 6124 |
| **Imputed hs-cTnT dataset** | | | |
| **Control** | 45 | 30857 | 7152 |
| **RIPC** | 43 | 22809 | 10704 |
| **Nitrates** | 48 | 35350 | 13090 |
| **RIPC+Nitrates** | 49 | 32831 | 11284 |

RIPC, Remote ischaemic preconditioning; SEM, standard error of mean

**Supplementary Table 2. Patient characteristics, by RIPC and nitrates (either intraoperative IV GTN or long-lasting oral nitrates) for ERICCA post-hoc analysis**

|  | **Control** | | **RIPC alone** | | **Nitrates alone** | | **RIPC + Nitrates** | |
| --- | --- | --- | --- | --- | --- | --- | --- | --- |
|  |  |  |  |  |  |  |  |  |
|  |  |  |  |  |  |  |  |  |
| **Total patients with data** | 446 | | 445 | | 308 | | 303 | |
|  |  |  |  |  |  |  |  |  |
| **Ethnicity - N (%)** |  |  |  |  |  |  |  |  |
| White | 423 | (94.8) | 420 | (94.4) | 292 | (94.8) | 286 | (94.4) |
| Asian | 21 | (4.7) | 23 | (5.2) | 13 | (4.2) | 11 | (3.6) |
| Black | 2 | (0.4) | 0 | (0.0) | 2 | (0.6) | 4 | (1.3) |
| Other | 0 | (0.0) | 2 | (0.4) | 1 | (0.3) | 2 | (0.7) |
| **NYHA class - N (%)** |  |  |  |  |  |  |  |  |
| None | 69 | (15.5) | 78 | (17.5) | 47 | (15.3) | 39 | (12.9) |
| I | 57 | (12.8) | 64 | (14.4) | 40 | (13.0) | 39 | (12.9) |
| II | 185 | (41.5) | 182 | (40.9) | 126 | (40.9) | 140 | (46.2) |
| III | 130 | (29.1) | 110 | (24.7) | 88 | (28.6) | 79 | (26.1) |
| IV | 5 | (1.1) | 11 | (2.5) | 7 | (2.3) | 6 | (2.0) |
| **CCS angina class - N (%)** |  |  |  |  |  |  |  |  |
| None | 155 | (34.8) | 134 | (30.1) | 82 | (26.6) | 63 | (20.8) |
| I | 84 | (18.8) | 73 | (16.4) | 47 | (15.3) | 40 | (13.2) |
| II | 112 | (25.1) | 144 | (32.4) | 100 | (32.5) | 114 | (37.6) |
| III | 75 | (16.8) | 61 | (13.7) | 49 | (15.9) | 51 | (16.8) |
| IV | 20 | (4.5) | 33 | (7.4) | 30 | (9.7) | 35 | (11.6) |
| **LVEF - N (%)** |  |  |  |  |  |  |  |  |
| Normal/good | 286 | (67.0) | 282 | (67.1) | 195 | (66.6) | 183 | (64.9) |
| Moderate | 91 | (21.3) | 89 | (21.2) | 59 | (20.1) | 70 | (24.8) |
| Poor | 50 | (11.7) | 49 | (11.7) | 39 | (13.3) | 29 | (10.3) |
| N (%) Missing | 19 | (4.3) | 25 | (5.6) | 15 | (4.9) | 21 | (6.9) |
| **Number of diseased vessels - N (%)** |  |  |  |  |  |  |  |  |
| 1 | 93 | (20.9) | 79 | (17.8) | 40 | (13.0) | 39 | (12.9) |
| 2 | 91 | (20.4) | 94 | (21.1) | 77 | (25.0) | 65 | (21.5) |
| 3 | 262 | (58.7) | 272 | (61.1) | 191 | (62.0) | 199 | (65.7) |
| **Creatinine (μmol/L)** |  |  |  |  |  |  |  |  |
| Mean (SEM) | 94.7 | (1.6) | 93.5 | (1.3) | 94.8 | (2.2) | 95.3 | (1.5) |
| Range (Min, Max) |  | [45, 564] |  | [39, 196] |  | [46, 569] |  | [42, 177] |

Notes: N, number; %, percentage; NYHA, New York Heart Association; CCS, Canadian Cardiovascular Society; LVEF, left ventricular ejection fraction; SEM, standard error of mean.

**Supplementary Table 3. Medication use at baseline, by RIPC and nitrates (either intraoperative IV GTN or long-lasting oral nitrates) for ERICCA post-hoc analysis**

|  | **Control** | | **RIPC alone** | | **Nitrates alone** | | **RIPC + Nitrates** | |
| --- | --- | --- | --- | --- | --- | --- | --- | --- |
|  |  |  |  |  |  |  |  |  |
|  |  |  |  |  |  |  |  |  |
| **Total patients with data** | 446 | | 445 | | 308 | | 303 | |
|  |  |  |  |  |  |  |  |  |
| **Aspirin - N (%)** | 350 | (78.5) | 350 | (78.7) | 254 | (82.5) | 250 | (82.5) |
| **Beta-blocker - N (%)** | 286 | (64.1) | 282 | (63.4) | 202 | (65.6) | 209 | (69.0) |
| **Calcium channel blocker - N (%)** | 122 | (27.4) | 125 | (28.1) | 87 | (28.2) | 104 | (34.3) |
| **Cholesterol lowering - N (%)** | 388 | (87.0) | 367 | (82.5) | 267 | (86.7) | 265 | (87.5) |
| **ACE inhibitor - N (%)** | 259 | (58.1) | 280 | (62.9) | 190 | (61.7) | 200 | (66.0) |
| **Insulin - N (%)** | 30 | (6.7) | 26 | (5.8) | 25 | (8.1) | 23 | (7.6) |
| **Sulphonylurea - N (%)** | 26 | (5.8) | 17 | (3.8) | 23 | (7.5) | 25 | (8.3) |
| **Metformin - N (%)** | 77 | (17.3) | 73 | (16.4) | 50 | (16.2) | 54 | (17.8) |
| **Clopidogrel/Prasugrel - N (%)** | 99 | (22.2) | 111 | (24.9) | 70 | (22.7) | 86 | (28.4) |
| **Warfarin - N (%)** | 51 | (11.4) | 48 | (10.8) | 38 | (12.3) | 37 | (12.2) |
| **Diuretics - N (%)** | 171 | (38.3) | 166 | (37.3) | 136 | (44.2) | 107 | (35.3) |
|  |  |  |  |  |  |  |  |  |

Notes: N, number; %, percentage.

**Supplementary Table 4. Effect of RIPC and intraoperative IV GTN on all-cause mortality up to 12 months following surgery for ERICCA post-hoc analysis**

|  | **Unadjusted*** | | | | **Adjusted**** | | | |
| --- | --- | --- | --- | --- | --- | --- | --- | --- |
|  | **HR** | **95% CI** | | **p-value** | **HR** | **95% CI** | | **p-value** |
|  |  |  |  |  |  |  |  |  |
|  |  |  |  |  |  |  |  |  |
| **Interaction Nitrates*RIPC** | 1.77 | 0.79 | 3.94 | 0.165 | 1.84 | 0.83 | 4.11 | 0.135 |
|  |  |  |  |  |  |  |  |  |
| **Effect of RIPC in strata:** |  |  |  |  |  |  |  |  |
| No nitrates | 1.09 | 0.69 | 1.73 | 0.705 | 1.17 | 0.74 | 1.85 | 0.503 |
| Nitrates | 1.93 | 1.00 | 3.73 | 0.050 | 2.16 | 1.11 | 4.19 | 0.023 |
|  |  |  |  |  |  |  |  |  |
| **Comparison of each combination** |  |  |  |  |  |  |  |  |
| Sham RIPC, no nitrates (ref) |  |  |  |  |  |  |  |  |
| RIPC alone | 1.09 | 0.69 | 1.73 | 0.705 | 1.17 | 0.74 | 1.85 | 0.503 |
| Nitrates alone | 0.96 | 0.45 | 2.03 | 0.916 | 1.04 | 0.49 | 2.21 | 0.913 |
| RIPC + Nitrates | 1.85 | 0.95 | 3.60 | 0.069 | 2.25 | 1.14 | 4.43 | 0.019 |
|  |  |  |  |  |  |  |  |  |

Notes: HR, hazard ratio; CI, confidence interval.

* Cox Proportional hazards model with stratification by study site; ** Cox Proportional hazards model with stratification by study site and adjustment for EuroSCORE, body mass index (BMI), previous myocardial infarction, and diabetes.

**Supplementary Table 5: Effect of RIPC and intraoperative IV GTN on cardiovascular mortality up to 12 months following surgery for ERICCA post-hoc analysis**

|  | **Unadjusted*** | | | | **Adjusted**** | | | |
| --- | --- | --- | --- | --- | --- | --- | --- | --- |
|  | **HR** | **95% CI** | | **p-value** | **HR** | **95% CI** | | **p-value** |
|  |  |  |  |  |  |  |  |  |
|  |  |  |  |  |  |  |  |  |
| **Interaction Nitrates*RIPC** | 2.10 | 0.69 | 6.40 | 0.190 | 2.15 | 0.71 | 6.53 | 0.177 |
|  |  |  |  |  |  |  |  |  |
| **Effect of RIPC in strata:** |  |  |  |  |  |  |  |  |
| No nitrates | 1.27 | 0.72 | 2.25 | 0.413 | 1.38 | 0.78 | 2.45 | 0.274 |
| Nitrates | 2.67 | 1.03 | 6.92 | 0.043 | 2.96 | 1.14 | 7.70 | 0.026 |
|  |  |  |  |  |  |  |  |  |
| **Comparison of each combination** |  |  |  |  |  |  |  |  |
| Sham RIPC, no nitrates (ref) |  |  |  |  |  |  |  |  |
| RIPC alone | 1.27 | 0.72 | 2.25 | 0.413 | 1.38 | 0.78 | 2.45 | 0.274 |
| Nitrates alone | 0.62 | 0.22 | 1.75 | 0.366 | 0.69 | 0.25 | 1.95 | 0.486 |
| RIPC + Nitrates | 1.66 | 0.72 | 3.80 | 0.232 | 2.05 | 0.88 | 4.79 | 0.097 |
|  |  |  |  |  |  |  |  |  |

Notes: HR, hazard ratio; CI, confidence interval.

* Cox Proportional hazards model with stratification by study site; ** Cox Proportional hazards model with stratification by study site and adjustment for EuroSCORE , body mass index (BMI), previous myocardial infarction, and diabetes.

**Supplementary Table 6 Effect of RIPC and intraoperative IV GTN on perioperative myocardial infarction for ERICCA post-hoc analysis**

|  | **Unadjusted** | | | | **Adjusted*** | | | |
| --- | --- | --- | --- | --- | --- | --- | --- | --- |
|  | **OR** | **95% CI** | | **p-value** | **OR** | **95% CI** | | **p-value** |
|  |  |  |  |  |  |  |  |  |
|  |  |  |  |  |  |  |  |  |
| **Interaction Nitrates*RIPC** | 1.54 | 0.88 | 2.68 | 0.129 | 1.56 | 0.89 | 2.72 | 0.120 |
|  |  |  |  |  |  |  |  |  |
| **Effect of RIPC in strata:** |  |  |  |  |  |  |  |  |
| No nitrates | 0.77 | 0.57 | 1.03 | 0.078 | 0.77 | 0.57 | 1.03 | 0.080 |
| Nitrates | 1.18 | 0.74 | 1.88 | 0.491 | 1.19 | 0.75 | 1.91 | 0.462 |
|  |  |  |  |  |  |  |  |  |
| **Comparison of each combination** |  |  |  |  |  |  |  |  |
| Sham RIPC, no nitrates (ref) |  |  |  |  |  |  |  |  |
| RIPC alone | 0.77 | 0.57 | 1.03 | 0.078 | 0.77 | 0.57 | 1.03 | 0.080 |
| Nitrates alone | 0.74 | 0.47 | 1.16 | 0.184 | 0.74 | 0.47 | 1.18 | 0.206 |
| RIPC + Nitrates | 0.87 | 0.56 | 1.34 | 0.523 | 0.89 | 0.57 | 1.38 | 0.601 |
|  |  |  |  |  |  |  |  |  |

Notes: OR, odds ratio; CI, confidence interval.

* Adjusted for EuroSCORE, body mass index (BMI), previous myocardial infarction, and diabetes.

**Supplementary Table 7 Effect of RIPC and intraoperative IV GTN on peri-operative myocardial injury for ERICCA post-hoc analysis**

|  | **Unadjusted#** | | | | **Adjusted#*** | | | |
| --- | --- | --- | --- | --- | --- | --- | --- | --- |
|  | **Ratio** | **95% CI** | | **p-value** | **Ratio** | **95% CI** | | **p-value** |
|  |  |  |  |  |  |  |  |  |
|  |  |  |  |  |  |  |  |  |
| **Interaction nitrates*RIPC** | 1.04 | 0.88 | 1.23 | 0.622 | 1.05 | 0.89 | 1.24 | 0.576 |
|  |  |  |  |  |  |  |  |  |
| **Effect of RIPC in strata:** |  |  |  |  |  |  |  |  |
| No nitrates | 0.95 | 0.85 | 1.05 | 0.307 | 0.95 | 0.86 | 1.05 | 0.333 |
| Nitrates | 0.99 | 0.87 | 1.13 | 0.864 | 1.00 | 0.87 | 1.14 | 0.963 |
|  |  |  |  |  |  |  |  |  |
| **Comparison of each combination** |  |  |  |  |  |  |  |  |
| Sham RIPC, no nitrates (ref) |  |  |  |  |  |  |  |  |
| RIPC alone | 0.95 | 0.85 | 1.05 | 0.307 | 0.95 | 0.86 | 1.05 | 0.333 |
| Nitrates alone | 0.97 | 0.85 | 1.11 | 0.692 | 0.98 | 0.86 | 1.12 | 0.822 |
| RIPC + Nitrates | 0.96 | 0.84 | 1.10 | 0.570 | 0.98 | 0.86 | 1.12 | 0.785 |
|  |  |  |  |  |  |  |  |  |

Notes: CI, confidence interval. Ratio, ratio of geometric mean 72-h AUC hs-TnT. #Results using multiple imputation dataset.

* Adjusted for EuroSCORE, body mass index (BMI), previous myocardial infarction, and diabetes.

**Supplementary Table 8 Effect of RIPC, intraoperative IV GTN and long-lasting oral nitrates on all-cause mortality up to 12 months following surgery for ERICCA post-hoc analysis**

|  | Unadjusted* | | | | Adjusted** | | | |
| --- | --- | --- | --- | --- | --- | --- | --- | --- |
|  | HR | 95% CI | | p-value | HR | 95% CI | | p-value |
|  |  |  |  |  |  |  |  |  |
|  |  |  |  |  |  |  |  |  |
| **Interaction IV Nitrates*RIPC** | 2.09 | 0.82 | 5.35 | 0.125 | 2.21 | 0.86 | 5.67 | 0.098 |
| **Interaction oral Nitrates*RIPC** | 6.64 | 1.31 | 33.60 | 0.022 | 6.58 | 1.29 | 33.52 | 0.023 |
| **Interaction IV+oral Nitrates*RIPC** | 2.47 | 0.68 | 9.01 | 0.171 | 2.63 | 0.71 | 9.70 | 0.147 |
|  |  |  |  |  |  |  |  |  |
| **Effect of RIPC in strata:** |  |  |  |  |  |  |  |  |
| No nitrates | 0.85 | 0.52 | 1.40 | 0.524 | 0.90 | 0.55 | 1.50 | 0.695 |
| IV Nitrates | 1.77 | 0.80 | 3.93 | 0.157 | 2.00 | 0.90 | 4.44 | 0.089 |
| oral Nitrates | 5.64 | 1.21 | 26.27 | 0.027 | 5.95 | 1.27 | 27.84 | 0.023 |
| IV+oral Nitrates | 2.10 | 0.64 | 6.92 | 0.223 | 2.38 | 0.71 | 7.95 | 0.161 |
|  |  |  |  |  |  |  |  |  |
| **Comparison of each combination** |  |  |  |  |  |  |  |  |
| **Sham RIPC, no nitrates (ref)** |  |  |  |  |  |  |  |  |
| RIPC alone | 0.85 | 0.52 | 1.40 | 0.524 | 0.90 | 0.55 | 1.50 | 0.695 |
| IV nitrates alone | 0.83 | 0.36 | 1.89 | 0.656 | 0.89 | 0.39 | 2.04 | 0.781 |
| Oral nitrates alone | 0.41 | 0.10 | 1.74 | 0.230 | 0.42 | 0.10 | 1.78 | 0.239 |
| IV+oral nitrates | 1.62 | 0.51 | 5.12 | 0.409 | 1.83 | 0.58 | 5.80 | 0.304 |
| RIPC+IV nitrates | 1.47 | 0.71 | 3.04 | 0.296 | 1.78 | 0.85 | 3.72 | 0.127 |
| RIPC+oral nitrates | 2.34 | 1.11 | 4.94 | 0.026 | 2.50 | 1.18 | 5.30 | 0.017 |
| RIPC+IV+oral nitrates | 9.16 | 1.32 | 63.39 | 0.025 | 10.89 | 1.58 | 74.92 | 0.015 |
|  |  |  |  |  |  |  |  |  |

Notes: HR, hazard ratio; CI, confidence interval.

* Cox Proportional hazards model with stratification by study site; ** Cox Proportional hazards model with stratification by study site and adjustment for EuroSCORE, body mass index (BMI), previous myocardial infarction, and diabetes.

**Supplementary Table 9 Effect of RIPC, intraoperative IV GTN and long-lasting oral nitrates on cardiovascular mortality up to 12 months following surgery for ERICCA post-hoc analysis**

|  | Unadjusted* | | | | Adjusted** | | | |
| --- | --- | --- | --- | --- | --- | --- | --- | --- |
|  | HR | 95% CI | | p-value | HR | 95% CI | | p-value |
|  |  |  |  |  |  |  |  |  |
|  |  |  |  |  |  |  |  |  |
| **Interaction IV Nitrates*RIPC** | 2.56 | 0.67 | 9.76 | 0.168 | 2.66 | 0.70 | 10.13 | 0.153 |
| **Interaction oral Nitrates*RIPC** | 8.14 | 0.89 | 74.30 | 0.063 | 7.21 | 0.78 | 66.50 | 0.081 |
| **Interaction IV+oral Nitrates*RIPC** | 2.62 | 0.46 | 14.76 | 0.275 | 2.54 | 0.44 | 14.50 | 0.294 |
|  |  |  |  |  |  |  |  |  |
| **Effect of RIPC in strata:** |  |  |  |  |  |  |  |  |
| No nitrates | 0.98 | 0.53 | 1.82 | 0.946 | 1.07 | 0.57 | 1.99 | 0.843 |
| IV Nitrates | 2.51 | 0.77 | 8.18 | 0.127 | 2.83 | 0.86 | 9.27 | 0.086 |
| oral Nitrates | 7.97 | 0.96 | 66.25 | 0.055 | 7.68 | 0.92 | 64.32 | 0.060 |
| IV+oral Nitrates | 2.56 | 0.51 | 12.85 | 0.252 | 2.71 | 0.53 | 13.79 | 0.231 |
|  |  |  |  |  |  |  |  |  |
| **Comparison of each combination** |  |  |  |  |  |  |  |  |
| **Sham RIPC, no nitrates (ref)** |  |  |  |  |  |  |  |  |
| RIPC alone | 0.98 | 0.53 | 1.82 | 0.946 | 1.07 | 0.57 | 1.99 | 0.843 |
| IV nitrates alone | 0.51 | 0.16 | 1.66 | 0.261 | 0.55 | 0.17 | 1.82 | 0.329 |
| Oral nitrates alone | 0.36 | 0.05 | 2.70 | 0.319 | 0.38 | 0.05 | 2.87 | 0.347 |
| IV+oral nitrates | 1.27 | 0.26 | 6.20 | 0.770 | 1.55 | 0.32 | 7.60 | 0.589 |
| RIPC+IV nitrates | 1.27 | 0.51 | 3.21 | 0.607 | 1.57 | 0.61 | 4.02 | 0.351 |
| RIPC+oral nitrates | 2.86 | 1.14 | 7.18 | 0.025 | 2.91 | 1.15 | 7.32 | 0.024 |
| RIPC+IV+oral nitrates | 10.10 | 0.70 | 145.08 | 0.089 | 11.90 | 0.83 | 170.22 | 0.068 |
|  |  |  |  |  |  |  |  |  |

Notes: HR, hazard ratio; CI, confidence interval.

* Cox Proportional hazards model with stratification by study site; ** Cox Proportional hazards model with stratification by study site and adjustment for EuroSCORE, body mass index (BMI), previous myocardial infarction, and diabetes.

**Supplementary Table 10: Effect of RIPC, intraoperative IV GTN and long-lasting oral nitrates on perioperative myocardial infarction for ERICCA post-hoc analysis**

|  | Unadjusted | | | | Adjusted** | | | |
| --- | --- | --- | --- | --- | --- | --- | --- | --- |
|  | HR | 95% CI | | p-value | HR | 95% CI | | p-value |
|  |  |  |  |  |  |  |  |  |
|  |  |  |  |  |  |  |  |  |
| **Interaction IV nitrates*RIPC** | 1.60 | 0.88 | 2.90 | 0.125 | 1.63 | 0.89 | 2.97 | 0.111 |
| **Interaction oral nitrates*RIPC** | 2.09 | 0.86 | 5.09 | 0.103 | 2.13 | 0.87 | 5.21 | 0.096 |
| **Interaction IV+oral nitrates*RIPC** | 2.69 | 0.70 | 10.34 | 0.150 | 2.62 | 0.68 | 10.12 | 0.163 |
|  |  |  |  |  |  |  |  |  |
| **Effect of RIPC in strata:** |  |  |  |  |  |  |  |  |
| No nitrates | 0.70 | 0.51 | 0.96 | 0.025 | 0.69 | 0.50 | 0.95 | 0.025 |
| IV Nitrates | 1.11 | 0.67 | 1.84 | 0.687 | 1.13 | 0.68 | 1.88 | 0.638 |
| oral Nitrates | 1.46 | 0.64 | 3.33 | 0.373 | 1.48 | 0.64 | 3.39 | 0.355 |
| IV+oral Nitrates | 1.87 | 0.51 | 6.92 | 0.349 | 1.81 | 0.49 | 6.75 | 0.374 |
|  |  |  |  |  |  |  |  |  |
| **Comparison of each combination** |  |  |  |  |  |  |  |  |
| **Sham RIPC, no nitrates (ref)** |  |  |  |  |  |  |  |  |
| RIPC alone | 0.70 | 0.51 | 0.96 | 0.025 | 0.69 | 0.50 | 0.95 | 0.025 |
| IV nitrates alone | 0.75 | 0.47 | 1.21 | 0.243 | 0.76 | 0.47 | 1.22 | 0.249 |
| Oral nitrates alone | 0.63 | 0.33 | 1.20 | 0.158 | 0.64 | 0.33 | 1.22 | 0.177 |
| IV+oral nitrates | 0.40 | 0.13 | 1.20 | 0.101 | 0.44 | 0.14 | 1.33 | 0.145 |
| RIPC+IV nitrates | 0.84 | 0.53 | 1.33 | 0.453 | 0.85 | 0.53 | 1.36 | 0.508 |
| RIPC+oral nitrates | 0.91 | 0.50 | 1.67 | 0.768 | 0.95 | 0.51 | 1.74 | 0.857 |
| RIPC+IV+oral nitrates | 0.58 | 0.15 | 2.30 | 0.438 | 0.65 | 0.16 | 2.61 | 0.542 |
|  |  |  |  |  |  |  |  |  |

Notes: OR, odds ratio; CI, confidence interval.

* Adjusted for EuroSCORE, body mass index (BMI), previous myocardial infarction, and diabetes.

**Supplementary Table 11 Effect of RIPC, intraoperative IV GTN and long-lasting oral nitrates on peri-operative myocardial injury for ERICCA post-hoc analysis**

|  | Unadjusted# | | | | Adjusted#* | | | |
| --- | --- | --- | --- | --- | --- | --- | --- | --- |
|  | Ratio | 95% CI | | p-value | Ratio | 95% CI | | p-value |
|  |  |  |  |  |  |  |  |  |
|  |  |  |  |  |  |  |  |  |
| **Interaction IV Nitrates*RIPC** | 1.06 | 0.89 | 1.27 | 0.492 | 1.08 | 0.90 | 1.29 | 0.412 |
| **Interaction oral Nitrates*RIPC** | 1.14 | 0.87 | 1.50 | 0.349 | 1.17 | 0.89 | 1.54 | 0.259 |
| **Interaction IV+oral Nitrates*RIPC** | 1.10 | 0.78 | 1.54 | 0.591 | 1.08 | 0.78 | 1.51 | 0.643 |
|  |  |  |  |  |  |  |  |  |
| **Effect of RIPC in strata:** |  |  |  |  |  |  |  |  |
| No nitrates | 0.93 | 0.83 | 1.03 | 0.160 | 0.93 | 0.84 | 1.03 | 0.157 |
| IV Nitrates | 0.99 | 0.85 | 1.14 | 0.865 | 1.00 | 0.86 | 1.16 | 0.991 |
| oral Nitrates | 1.06 | 0.82 | 1.37 | 0.671 | 1.09 | 0.84 | 1.40 | 0.530 |
| IV+oral Nitrates | 1.02 | 0.74 | 1.40 | 0.917 | 1.00 | 0.73 | 1.38 | 0.981 |
|  |  |  |  |  |  |  |  |  |
| **Comparison of each combination** |  |  |  |  |  |  |  |  |
| **Sham RIPC, no nitrates (ref)** |  |  |  |  |  |  |  |  |
| RIPC alone | 0.93 | 0.83 | 1.03 | 0.160 | 0.93 | 0.84 | 1.03 | 0.157 |
| IV nitrates alone | 0.97 | 0.84 | 1.11 | 0.638 | 0.97 | 0.85 | 1.12 | 0.696 |
| Oral nitrates alone | 0.89 | 0.74 | 1.08 | 0.234 | 0.90 | 0.75 | 1.09 | 0.290 |
| IV+oral nitrates | 0.89 | 0.69 | 1.15 | 0.390 | 0.95 | 0.74 | 1.23 | 0.709 |
| RIPC+IV nitrates | 0.95 | 0.83 | 1.10 | 0.518 | 0.97 | 0.85 | 1.12 | 0.688 |
| RIPC+oral nitrates | 0.94 | 0.77 | 1.16 | 0.572 | 0.98 | 0.80 | 1.20 | 0.856 |
| RIPC+IV+oral nitrates | 0.91 | 0.71 | 1.16 | 0.449 | 0.96 | 0.75 | 1.22 | 0.719 |
|  |  |  |  |  |  |  |  |  |

Notes: CI, confidence interval. Ratio, ratio of geometric mean 72-hour AUC hs-TnT. #Results using multiple imputation dataset.

* Adjusted for EuroSCORE, body mass index (BMI), previous myocardial infarction, and diabetes.

**Supplementary Figure 1. Kaplan-Meier estimate of cumulative incidence of mortality, by RIPC and intraoperative IV GTN for ERICCA post-hoc analysis**

**Supplementary Figure 2. Kaplan-Meier estimate of cumulative incidence of cardiovascular mortality, by RIPC and intraoperative IV GTN for ERICCA post-hoc analysis**

**Supplementary Figure 3. Kaplan-Meier estimate of cumulative incidence of mortality, by RIPC, intraoperative IV GTN and long-lasting oral nitrates for ERICCA post-hoc analysis**

**Supplementary Figure 4. Kaplan-Meier estimate of cumulative incidence of cardiovascular mortality, by RIPC, intraoperative IV GTN and long-lasting oral nitrates for ERICCA post-hoc analysis**
